# Doctors in Parliament

**Published:** 1903-07-11

**Authors:** 


### The Hospital.

A Journal of

The Medical Sciences and Hospital Administration.

Vol. XXXIV.—No. 876. Edited by Sir Henry Burdett, K.C.B. [July 11, 1903.

### Doctors in Parliament.

A Statement has lately appeared in several papers, resting upon we know not what foundation, to the effect that Sir Frederick Treves is about to retire from practice, and that he intends to seek election into the House of Commons. If the first part of the report be true, we hope that the second part may be true also; because there is no other way in which Sir Frederick could so effectively minimise the loss to the public which must necessarily attend upon his relinquishment of professional activity. It is trite to observe that the legislation of the present day deals with an increasing number of questions for the satisfactory solution of which medical knowledge, or perhaps more strictly physiological knowledge, is urgently required; and as to which such knowledge would be cordially welcomed by Parliament, if it were supplied by any member of their own body in whose accuracy and sagacity they had learnt to trust, and who was recognised as an exponent of scientific belief as distinguished from political opinion. The absence of the last-mentioned qualification has, we imagine, seriously diminished the influence which might otherwise have been exerted by two of the present medical members of the House, Sir Walter Foster and Dr. Farquharson, both of whom have come to be looked upon primarily as politicians and only secondarily as doctors. Both would be suspected, we think, by the man in the street, of addressing themselves to the discovery of medical grounds on which to support the views of their party, rather than to the application of any light which medical science might really be calculated to throw upon the question at issue; and this view, however unfounded it might be in any particular instance, and however unjust generally, cannot but militate seriously against the influence which these gentlemen, regarded simply as doctors, ought to be in a position to exert. Sir Frederick Treves would undoubtedly be welcomed by many constituencies, and it will be open to him, should he so decide, to occupy in the House a position of almost unique consideration and independence.

The problems which confront the generation now rising into complete strength and activity in this country are mainly two: the consolidation of the Empire into a self-contained and self-supporting aggregate of communities; and the rescue of the lower strata of the industrial classes from the conditions of filth and degradation in which they mostly live. The two are not only intimately connected together, but are practically interdependent; and the accomplishment of the second is only to be hoped for as a result of influences exerted gradually and persistently upon the young. What is humourously called "education" has been brought to the doors of the working classes of this country now for more than 30 years; and anyone who is curious about its effects may satisfy curiosity by a very simple expedient. It will be enough to get into a tramcar, say at the beginning of Theobald's Road, and to ride in it for a mile through the Clerkenwell district, observing the vehicle itself, and the manners, clothes, persons, and language of the passengers. A casual glance at the doors and windows of the news-shops in the streets will afford some idea of the class of literature which the operations of the School Board have brought into large demand; and will show that at least one chief desire of the local population is to obtain other people's money by gambling, that is to say, without honestly earning it. For any good purpose, the so-called education has wholly failed. It has not taught industry, it has not taught cleanliness, it has not taught decency of speech, it has not taught civility, it has not taught sobriety, it has not taught self-respect. It has failed to teach these things mainly because it has been engineered by sectarians for the purpose of teaching shibboleths which exert no influence upon life, but which still admit of being employed, with some effect, as battle-cries with which to rouse the worst passions of the ignorant and the debased. The recent and continuing opposition to the Education Act has been mainly called into existence by a very well-grounded fear lest it should render education a reality, and hence should emancipate the lower orders of people, from the control of the leaders whom they have been accustomed to obey. A result which is anticipated with terror by the ranting preacher, or by the trade-union spouter, should be the aim and hope of sensible and honest men; and we must in great measure look to the schools of the future to remedy evils which have been suffered to grow unchecked by those of the past. Education is a physiological process, which can only be rightly conducted under the guidance of physiological principles; and the same statement may be made with regard to all statutory provision for the better nurture or better training of the young. Parliament is now considering an Employment of Children Bill; and the two somewhat lengthy debates which have been held upon the report stage of this measure have been singularly fruitful in rhetoric uncontrolled by knowledge. The children of the lowest classes in our great towns may be rendered a source of strength to the nation, or may be permitted to become a source of perennial weakness and danger. In order to avoid the latter issue, and to secure the attainment of the former, the legislature greatly needs the help of men who are too firmly grounded in the truths of science to be led astray by the outcries of party politicians.

